# Supplementary material for: A Comparative Study of Genetic Responses to Short- and Long-Term Habitat Fragmentation in a Distylous Herb Hedyotis chyrsotricha (Rubiaceae)
Source: Plants (Basel). 2022 Jul 7;11(14):1800. doi: 10.3390/plants11141800 (PMC9323511; doi:10.3390/plants11141800)
Supplement: Supplementary file 1 [file plants-11-01800-s001.zip › Figure S1.pdf]

(A)

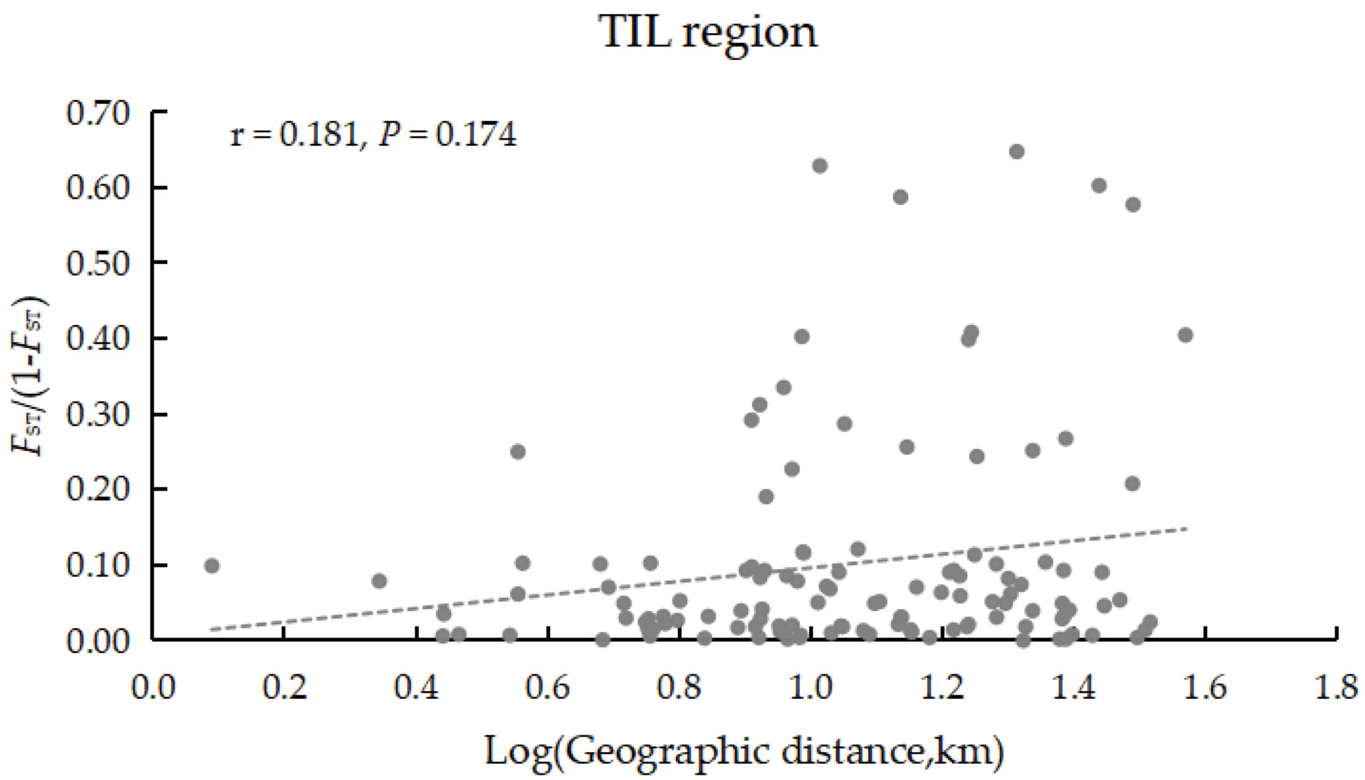

(B)

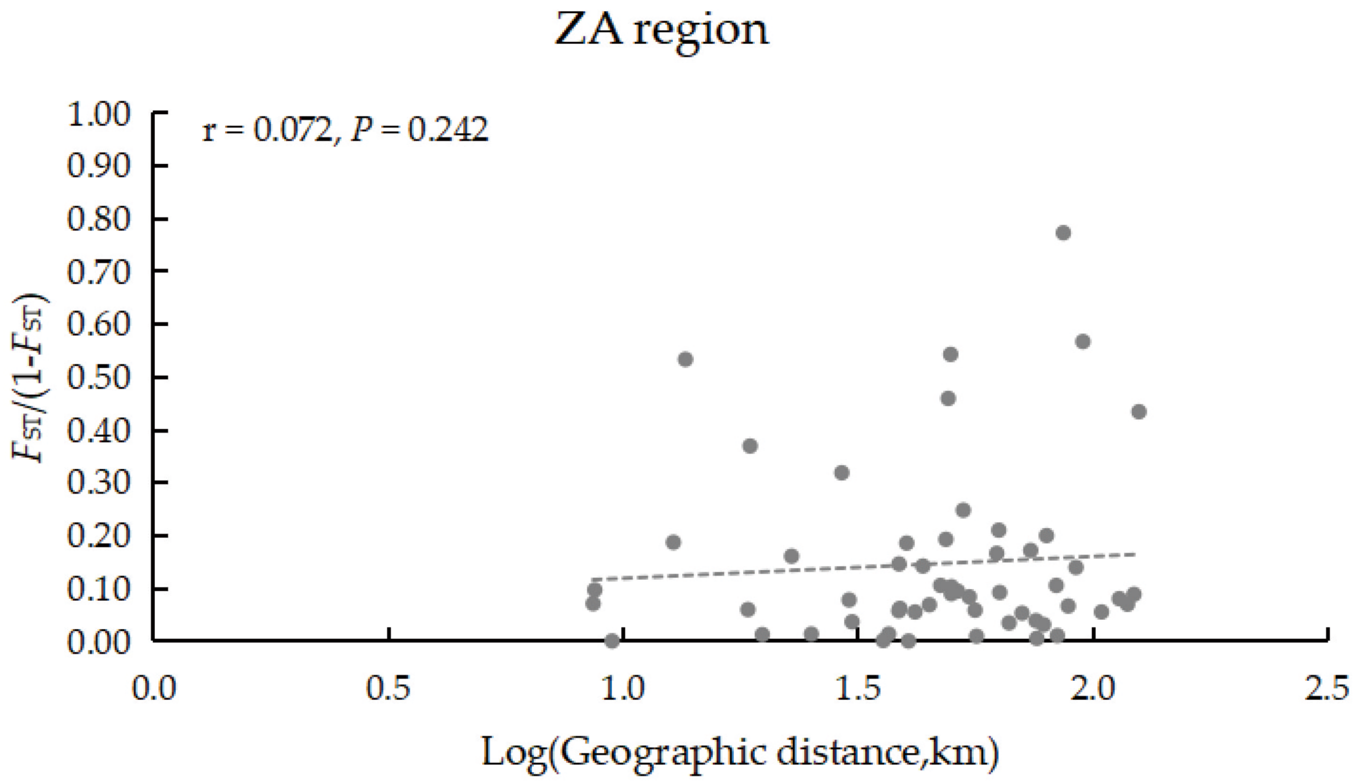

Figure S1. Analysis of isolation by distance based on FST/(1-FST) and the geographic distance (log10 transformed) in (A) the Thousand-Island Lake (TIL) region and (B) the Zhoushan Archi-pelago (ZA) region.
