# Supplementary figures and images for: A Comparative Study of Genetic Responses to Short- and Long-Term Habitat Fragmentation in a Distylous Herb Hedyotis chyrsotricha (Rubiaceae)
Source: Plants (Basel). 2022 Jul 7;11(14):1800. doi: 10.3390/plants11141800 (PMC9323511; doi:10.3390/plants11141800)

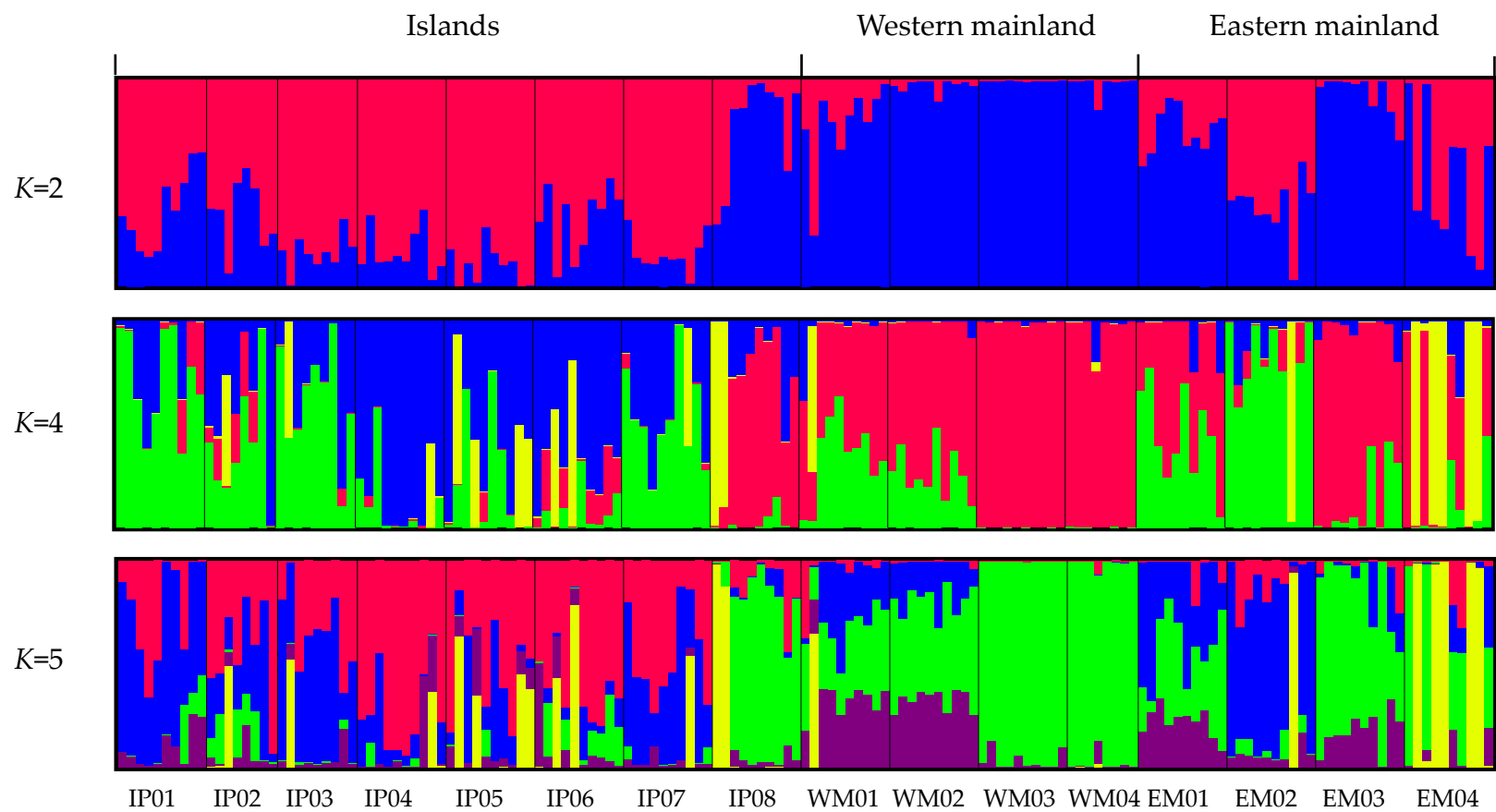

Supplement: Supplementary file 1 [file plants-11-01800-s001.zip › Figure S2.pdf]

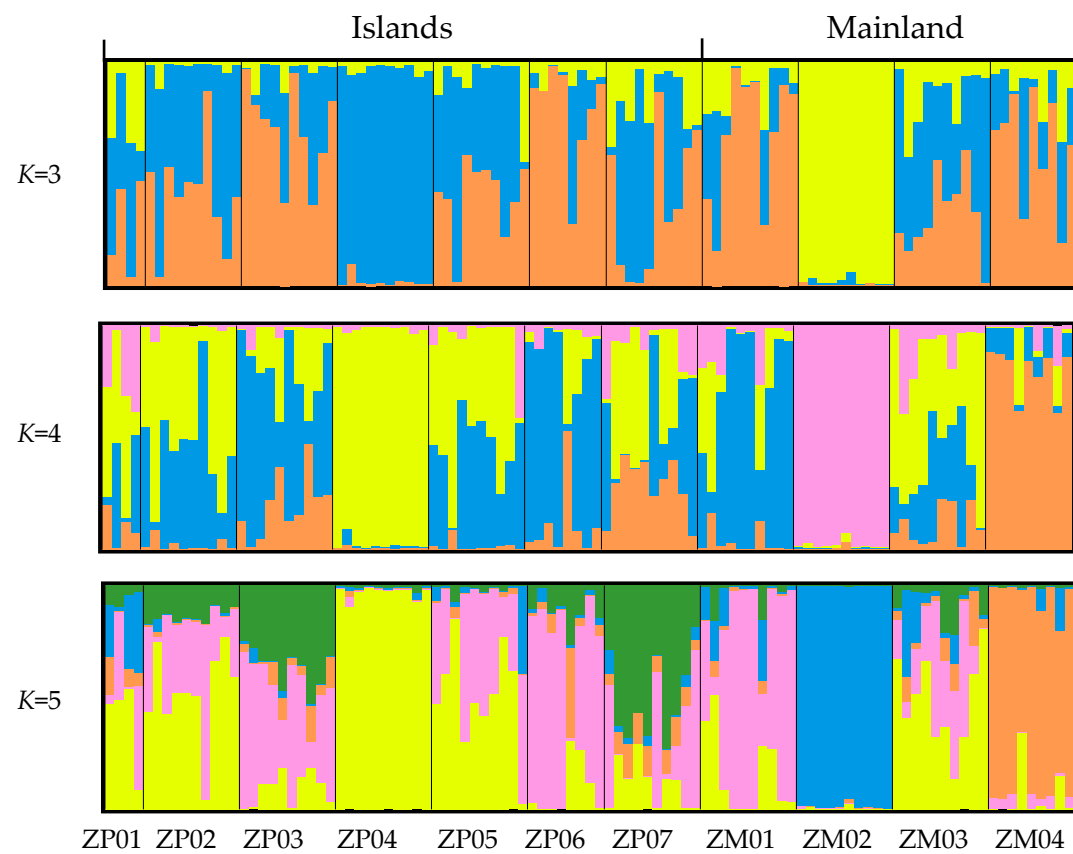

Supplement: Supplementary file 1 [file plants-11-01800-s001.zip › Figure S3.pdf]
